# Familiar Instructions in Medicine and Surgery, with Observations on the Means of Maintaining the Health of Men on Ship-Board, or When Employed in Unhealthy Localities. Intended for the Use of the Merchant Navy, Commanders of Yachts, Travellers, and All Persons Who May Be Away from Medical Aid

**Published:** 1847-07

**Authors:** 


### ART. XIII.

1. *Familiar Instructions in Medicine and Surgery, with Observations on the Means of maintaining the Health of Men on Ship-board, or when employed in Unhealthy Localities. Intended for the Use of the Merchant Navy, Commanders of Yachts, Travellers, and all Persons who may be away from Medical Aid.* By F. F. SANKEY, M.D.—*Malta,* 1846. 8vo, pp. 273.

2. *The Scale of Medicines with which Merchant Vessels are to be furnished, in pursuance of Act* 7 *and* 8 *Vic., cap.* 112*; with Directions for their Use; Observations on some of the Accidents and Diseases to which Seamen are more peculiarly liable; aud Directions for preserving the Health and promoting the Comfort of Merchant Seamen.* By CHARLES M'ARTHUR, M.D., Surgeon Royal Navy. Fourth Edition.—*London,* 1845. 8vo, pp. 76.

IN 1844 many respectable London merchants became impressed with the necessity of their ships being supplied with proper quantities of necessary medicines, and the masters of the ships with clear aud sufficient directions for their use, with information regarding the means to be employed after severe accidents. They accordingly applied to the Admiralty, and Dr. M'Arthur was appointed by Sir William Burnet, the Director-General of the Medical Department of the Navy, to draw up a scale of medicines with which every merchant ship not carrying a surgeon, was directed to be furnished by Act of Parliament. He also drew up the small work before us as a companion to the medicine chest. The fact of its having in one year reached a fourth edition is alone sufficient to show the demand which existed for some such work, and also, that it, to a certain extent, was calculated to supply that demand; but, on looking it over, we can readily believe, what we know to be the case, that many intelligent merchant captains have complained that it is not sufficiently full or detailed on the subjects treated, and that others on which they require information are altogether omitted. Dr. Sankey, another naval surgeon, practising at Malta, has endeavoured to avoid these objections in another very useful little work, the title of which is also given above.

Every ship which carries one hundred persons or upwards on board, and every ship having fifty persons or upwards on board, the voyage of which is likely to exceed twelve weeks, is obliged, by act of Parliament to carry as one of the complement, "some person duly authorized by law to practise in this kingdom as a physician, surgeon, or apothecary." But by far the greater proportion of our immense merchant navy do not fall within the provisions of this act, and therefore it is at once evident that the works before us are much more important than would at first sight appear, the commercial prosperity of our country so directly depending upon the health and welfare of our seamen. On these grounds we shall examine the works of Drs. Sankey and M'Arthur more closely than we are in the habit of examining works directed to the non-medical public.

It may not be uninteresting to our readers to look over the scale of medicines drawn up by Dr. M'Arthur, and sanctioned by act of Parliament. We therefore extract his table. The quantities are to be supplied for ten men, and to be increased proportionally to any additional number of men borne:

| | lbs. | oz. | drs. | | lbs. | oz. | drs. |
|---|---|---|---|---|---|---|---|
| Castor oil . . . | 1 | 0 | 0 | Balsam of capivi . . | 0 | 8 | 0 |
| Epsom salts . . . | 4 | 0 | 0 | Opodeldoc . . . | 0 | 8 | 0 |
| Powder of jalap . . | 0 | 4 | 0 | Spirit of hartshorn . . | 0 | 4 | 0 |
| rhubarb . . | 0 | 4 | 0 | Paregoric elixir . . | 0 | 4 | 0 |
| ginger . . | 0 | 2 | 0 | Essence of peppermint . | 0 | 1 | 0 |
| Carbonate of soda . . | 0 | 8 | 0 | Alum . . . | 0 | 8 | 0 |
| Tartaric acid . . | 0 | 8 | 0 | Blue stone . . . | 0 | 4 | 0 |
| Senna leaves . . . | 0 | 6 | 0 | Quinine . . . | 0 | 0 | 4 |
| Sulphur . . . | 0 | 8 | 0 | Calomel . . . | 0 | 4 | 0 |
| Cream of tartar . . | 0 | 8 | 0 | Dover's powder . . | 0 | 2 | 0 |
| Nitre . . . | 1 | 0 | 0 | Mustard . . . | 1 | 0 | 0 |
| Laudanum . . . | 0 | 4 | 0 | Mercurial ointment . . | 0 | 8 | 0 |
| Tincture of rhubarb . . | 0 | 8 | 0 | | | | |

"Bullin's blistering fluid, instead of blistering plaster; 2 yards of adhesive plaster; lint, 4 oz.; simple cerate, 8 oz.; basilicon ointment, 8 oz.

"*Pills.* 2 doz. Purg. (Pil. Col. Co. Ed. Phar.); 2 doz. Blue pills; 2 doz. Opium pills (Phar. Edin.)

"*Powders.* 2 doz. Purgative, each to contain ʒj of compound powder of Jalap and 2 grs. of Calomel; 2 doz. Emetic, each to contain 15 grs. of Ipecacuanha and 2 grs. of Tartrate of Antimony; 2 doz. Sudorific, each to contain 10 grs. of Nitre, 10 grs. of Cream of Tartar, and 5 grs. of Dover's powder; 2 doz. Injection, each to contain ʒss. of the Acetate of Zinc.

"1 set scales and weights; 1 marble mortar and pestle; 1 file; 1 graduated measure; 1 funnel; 1 small pewter cup; 2 pewter teaspoons; 1 spatula; 1 pair of scissors; 1 syringe; 2 lancets; 6 bandages of different sizes; 6 yards of calico; a paper of needles, pins, and thread.

"The pills and powders are to be carefully put up in tin or leaden boxes, and properly labelled."

Dr. Sankey has made the necessary additions of Diluted Sulphuric Acid, a Caustic Case, an Elastic Catheter, Enema Apparatus, Tourniquet, and a set of Splints for legs and arms. These are so obviously necessary that we are surprised they should have been overlooked in the official scale; but we cannot recommend some other additions of Dr. Sankey, such as Croton Oil, Creosote, James's Powder, Red Precipitate, Sugar of Lead, and Tincture of Colchicum, as it is of so much importance in a small ship only to take what is absolutely necessary, in order to avoid expense, confusion, and mistakes. Perhaps the Iodide of Potassium and the Muriated Tincture of Iron, in Dr. Sankey's list, might be advantageously admitted. For the reasons just stated, we also think the powdered Jalap, Senna Leaves, Tincture of Rhubarb, and Opodeldoc might be omitted from the official list. There are sufficient purgative medicines without the first two; a glass of wine and water with a little spice would answer every purpose of the kind; every ship carries some oil and turpentine, and with this a liniment can be immediately prepared equal if not superior to opodeldoc. Instead of these medicines we should increase the quantities of Cream of Tartar, Laudanum, and Quinine. Half an ounce of the latter is far from sufficient for a ship trading in tropical climates. Some cotton wool for burns, and oiled silk should also be added.

The first part of Dr. Sankey's work is a sort of medical dictionary, embracing the most important subjects which interest the parties he addresses. He has strictly avoided everything like pedantry or technicality, and has written in a most explicit and intelligible manner, adapting plain and familiar language with great tact to the common sense of his readers. The few pages in Dr. M'Arthur's work, on Accidents and Diseases, contain a great deal of very useful information in a very highly condensed form, but he has altogether omitted some important subjects; retention of urine, for example, is unnoticed, although particularly common among seamen; nor do we find any mention of hernia, catarrh, chilblains, whitlows, boils, piles, jaundice, itch, tooth- or ear-ache, all of which are of very frequent occurrence at sea. The language, also is somewhat technical, and by no means so intelligible to uneducated persons as that of Dr. Sankey. On the whole, we think the work of the latter by far the best of the kind which has hitherto appeared, but it contains some directions which might be amended.

The general directions for the suppresssion of bleeding from wounds are very good, and the course of the principal arteries, with the spots for the application of the pad of the tourniquet are shown by a plate, but we very much doubt the propriety of telling a sailor, "you would be justified in cutting down upon the vessel, enlarging the wound, and searching for the cut artery." (p. 11.)

A great deal of useful information is given regarding dislocations and fractures, but we think a few simple woodcuts would have been much more acceptable. We are not sure if any description would render the appearances produced by these accidents recognizable, while it would be very easy to point out that when a limb is in the position shown by one plate, it should be replaced by the means shown in another.

As a general rule, we entirely depart from the practice inculcated in fevers. With regard to letting of blood, we are told that "a decided impression should be made on the disease by the quantity removed; and the pulse should be reduced in force and frequency by the loss." (p. 69.) Dr. M'Arthur holds much the same opinions; but we maintain that such treatment is much more likely to do harm than good, even in the hands of a medical man. Experience is daily more strongly proving that fevers are the result of some specific poison, and run as determinate a course as smallpox, or any other specific disease, and that bleeding only lays the system more completely under the influence of the poison. Of course it will sometimes be a question with a medical man whether he had not better relieve some local symptom by depletion, even with the risk of depressing the general system; but we maintain that no such discretion should be given to a sailor. It would be far better—and generally it would be quite sufficient—to insist on thorough cleanliness, and ventilation, as much quiet as possible, cold sponging, simple diet, and plenty of water or bland fluid to drink. Neither of the writers before us have neglected to point out the importance of these matters, but in our opinion both have been much too heroic in the medical treatment they recommend a merchant captain to pursue—bleeding, emetics, purgatives, calomel, tartar-emetic, quinine, cold affusion, opium, wine, and blisters, forming a case of edge-tools, which would be far better kept out of sight.

Under the head of inflamed windpipe, the merchant captain is directed to "throw the head back to put the windpipe upon the stretch, and then just below the gristle or round ringbone of the throat under 'Adam's apple,' you begin to cut through the skin and flesh in a line down the centre for an inch; then push the point of the knife through into the windpipe, and make a cut in it, dividing two or three rings large enough to breathe freely through," (p. 160.) We fear, if this recommendation were attempted to be followed, we should have occasionally a literal "cutting the throat" in place of a sanative chirurgical operation.

In the treatment of wounds, Dr. Sankey leans a little to the seaman's weakness in favour of Friar's Balsam; but as he pours it *over* the bandage, it may perhaps be of use in forming a stiff covering to protect the wound from air and dirt. One merchant captain, to our knowledge, at once settled the fate of Dr. M'Arthur's scale and book by exclaiming "Why, sir, there's no Friar's Balsam in it!"

Dr. Sankey speaks very highly, and we believe with some reason, of the utility of an ointment, composed of a drachm of red precipitate and an ounce of resin cerate, in the indolent ulcers of seamen.

Both works contain very useful directions for preserving the health of seamen and avoiding disease, under the heads of food, clothing, bedding, cleanliness, ventilation, climate, intemperance, malaria, &c.; but we think that scales of diet in health and disease ought to have been given. Dr. M'Arthur speaks in high terms of the chloride of zinc introduced into use by Sir William Burnett, as a means of destroying the odour of bilge water, killing vermin, and preserving timber, canvas, cordage, and ship's stores. As it does not emit the disagreeable effluvia which form so strong an objection to the use of chloride of lime, nitrous gas, &c., it would be advisable to serve a regulated quantity with each medicine chest.

Dr. Sankey's book is concluded by an appendix containing a paper on Phthisis, some observations on Homœopathy and Hydropathy, with some useful general directions for travellers. As we have hitherto chiefly extracted what we considered objectionable, we think it but fair to give a specimen of the plain common sense of which the greater part of the book consists.

"*Homœopathy.* This new system of curing all diseases, is one of the novelties which occupy the world's attention for a time and make the reflective think. It is a plan to cure by influencing the imagination..............The medication by almost invisible globules ranks in the same class with fortune-telling and mesmerism. The conjuring part of the system by infinitesimal doses is founded on principles which are absurd and intolerable. But the manner of living to be observed by those who follow this cure is highly rational, and without which the doses of medicine would be utterly inert. [They are inert *with* the manner of living.] Persons are to abstain from all excesses, avoiding many things which experience proves to be hurtful, to live with great regularity as to hours and pleasures, to take air and exercise, to pass little time in bed, &c. &c. The whole treatment may be summed up in few words, 'To live moderately, and earn that living by your labour.' The cure of acute diseases can hardly be trusted to homœopathy. The nervous, the delicate, and the luxurious are the proper objects to be treated by this method. The system was invented by a man of genius, Hahnemann, and it has done infinite good by disabusing the world as to the necessity of employing in all cases powerful medicaments uncertain in their effects, rather than to assist Nature to remove the weight which is pressing her down. Every honest medical man must acknowledge that very many severe cases of disease are produced by impertinent interference, by rude medical discipline, and by the injurious exhibition of drugs; by which routine practitioners gain great reputation, first making the disease which they afterwards may have the merit of curing.

"No practitioner of any standing, but must be sensible of numerous errors of diagnosis made, and of ill-judged remedies prescribed, even by himself; errors which it has taken years of experience in the course of a long practice to correct. At any rate homœopathy is not so dangerous a weapon as physic in the hands of the ignorant, yet less effective when the latter is regulated by skill and judgment." (p. 81, pt. 2.)

We are rather surprised that our author, holding these opinions, should have recommended the violent means we have reprehended.

It will scarcely be believed, that in order to make the above extract readable, we have been obliged to omit two full stops, one colon, seven semicolons, and twenty-three commas, and this is by no means one of the worst specimens of punctuation in the book, which in this respect is perhaps the most extraordinary ever printed. The author makes an apology for "colonial getting up"—and well he may.

If a future edition of Dr. Sankey's work should be required, we would recommend an English publisher, and some of the alterations we have suggested, with separate lists of medicine for merchant vessels, yachtmen, and travellers, instead of one general list for these different classes. Scales of diet should also be given, and the use of plaster-of-Paris, or starch bandage in fracture described. Perhaps also, as women are often delivered on board ship, a few directions to be followed in cases of natural labour might be given; but we confess the subject to be a delicate one. The use of the probang also ought certainly to be described, as threatened suffocation from hastily swallowing large morsels of food is not an unfrequent occurrence, and requires the most prompt assistance.

We regard both works as well planned but imperfectly executed.

---

